# Endoscopic Detection of Early Esophageal Squamous Cell Carcinoma in Patients with Achalasia: Narrow-Band Imaging versus Lugol's Staining

**DOI:** 10.1155/2013/736756

**Published:** 2013-07-09

**Authors:** Edson Ide, Fred Olavo Aragão Andrade Carneiro, Mariana Souza Varella Frazão, Dalton Marques Chaves, Rubens Antônio Aissar Sallum, Eduardo Guimarães Hourneaux de Moura, Paulo Sakai, Ivan Cecconello, Fauze Maluf-Filho

**Affiliations:** ^1^Gastrointestinal Endoscopy Unit, São Paulo University Medical School, 05403-900 São Paulo, SP, Brazil; ^2^Digestive Surgery Department, São Paulo University Medical School, 05403-900 São Paulo, SP, Brazil; ^3^Endoscopy Unit, Cancer Institute of São Paulo University Medical School, 01246-000 São Paulo, SP, Brazil

## Abstract

Chromoendoscopy with Lugol's staining remains the gold standard technique for detecting superficial SCC. An alternative technique, such as narrow-band imaging (NBI), for “optical staining” would be desirable, since NBI is a simpler technique and has no known complications. In this study, we compare NBI without magnification and chromoendoscopy with Lugol's staining for detecting high-grade dysplasia and intramucosal esophageal squamous cell carcinoma (SCC) in patients with achalasia. This was a prospective observational study of 43 patients with achalasia referred to the Gastrointestinal Endoscopy Unit of the Hospital of Clinics, São Paulo, University Medical School, Brazil, from October 2006 to February 2007. Conventional examinations with white light, NBI, and Lugol staining were consecutively performed, and the suspected lesions were mapped, recorded, and sent for biopsy. The results of the three methods were compared regarding sensitivity, specificity, accuracy, positive predictive value, negative predictive value, positive likelihood value, and negative likelihood value. Of the 43 patients, one was diagnosed with esophageal squamous cell carcinoma, and it was detected by all of the methods. NBI technology without magnification has high sensitivity and negative predictive value for detecting superficial esophageal squamous cell carcinoma, and it has comparable results with those obtained with Lugol's staining.

## 1. Introduction

Achalasia is a chronic esophageal motility disorder associated with esophageal retention of foods and fluids, bacterial overgrowth, and impaired clearance of regurgitated gastric contents [1]. These factors usually lead to chronic inflammation of the esophageal mucosa, which potentially increases the risk of hyperplasia, dysplasia, and esophageal cancer [2, 3]. 

Esophageal squamous cell carcinomas in achalasia patients have been investigated previously. In a large cohort followup study, Wychulis et al. [4] analyzed 1,318 patients and found a 7-fold increased risk of esophageal squamous cell carcinomas in achalasia patients compared to the general population. Despite some contradictory data [5–7], achalasia is generally accepted as a condition associated with an increased risk for developing esophageal squamous cell carcinoma [8, 9].

Chromoendoscopy with Lugol's staining remains the gold standard technique for detecting superficial esophageal squamous cell carcinoma [10, 11]. Although Lugol's staining is a simple and low-cost method, instillation of its solution may lead to complications, such as hypersensitivity to iodine, laryngitis, and pneumonitis, as well as frequent painful sensations and nausea [12–15]. Kondo et al. [15] demonstrated a significant reduction in retrosternal discomfort with the use of sodium thiosulfate. An alternative technique such as narrow-band imaging for “optical staining” would be desirable, especially because it is a simpler technique and has no known complications.

Narrow-band imaging technology may be useful for detecting squamous cell carcinomas of the pharynx and esophagus. Muto et al. [16] and Yoshida et al. [17] observed morphological pattern changes in intrapapillary capillary loops. These patterns can be useful for diagnosing squamous cell carcinomas and even to predict lesion extension.

Various reports of early-stage pharyngeal and esophageal squamous cell carcinomas identified with narrow-band imaging technology can be found in the literature. Without using imaging magnification, Muto et al. [16] diagnosed a superficial squamous cell carcinoma of the pharynx, which appeared as a small and well-defined brownish area. Watanabe et al. [18], who also used narrow-band imaging without magnification, found six pharyngeal squamous cell carcinomas, and Goda et al. [19]   found an esophageal squamous cell carcinoma that was not identified by conventional endoscopy (obscure lesion). Recently, our group demonstrated that narrow-band imaging performs as well as Lugol's chromoendoscopy for the detection of esophageal squamous cell carcinoma in patients with head and neck cancer [20].

The aim of this study was to compare narrow-band imaging technology with Lugol's staining during endoscopic examination of the esophagus for the detection of high-grade intraepithelial neoplasias and superficial squamous cell carcinomas in patients with esophageal achalasia.

## 2. Materials and Methods

### 2.1. Design

This was a cross-sectional study, and esophageal mucosal examination was analyzed in a sequential approach divided into three phases. The first phase was with white light, the second phase was with narrow-band imaging, and the final phase was with Lugol's staining. At the end of each phase, abnormal findings were documented. 

The patients were submitted to an endoscopic procedure, and it was performed under conscious sedation with midazolam and fentanyl. Only one senior endoscopist (Edson Ide) performed all of the endoscopic procedures.

At the first phase, any residues or exudates were removed through water instillation. The esophagus was, then, analyzed with white light. If there were lesions, they were mapped using the anterior, posterior, right, and left esophageal walls and the distance up to anterior incisors as references. 

The second phase involved the use of narrow-band imaging assessment of the esophageal mucosa. At this moment, brownish areas were identified as lesions suspected of being neoplasia, compared to “normal” mucosa, which is green independent of changes in surface or vascular texture.

In the third phase, Lugol's staining was performed by spraying 20cc of a 2% Lugol's solution at esophageal mucosa. After the staining, white-colored areas were suspected of being neoplasia, in contrast with brown or brownish “normal” areas. 

The operator took biopsies of every suspicious lesion detected by any phase of the study. Biopsies were only performed after Lugol's staining was completed, and, after mucosal examination and biopsy, a volume of 20cc of a 0.5% sodium thiosulfate solution was instilled to remove Lugol's solution in order to reduce spasm and pain. Complications due to Lugol's solution were registered including laryngitis, chemical pneumonitis, hypersensitivity, and anaphylactic shock. 

The size and macroscopic shape of the lesions were evaluated according to the Paris Classification [21] for superficial esophageal lesions. Topography was divided into cervical (up to 5 cm of cricopharyngeus muscle), thoracic, and abdominal esophagus.

### 2.2. Patients

From October 2006 to February 2007, 48 consecutive patients with achalasia were referred to the Gastrointestinal Endoscopy Unit of a tertiary academic center for early esophageal cancer detection. These patients usually participate in a surveillance protocol, which consists of upper gastrointestinal endoscopy with associated esophageal Lugol's staining every 3 years.

Inclusion criteria were indications to take part in the surveillance protocol for patients with achalasia, despite any treatment (e.g., pharmacologic, endoscopic dilation, and surgical cardiomyotomy) they had undergone previously. Exclusion criteria were as follows: clinical conditions that prevented upper gastrointestinal endoscopy examination or Lugol's staining; previous history of allergic reaction to iodine; esophageal stasis that could not be cleared by endoscopic procedures; and endoscopic detection of an ulcerated, infiltrative, or stenotic lesion.

All participants provided written informed consent. This study was approved by the Ethics Committee of the University of São Paulo Medical School.

### 2.3. Endoscopy System

An Exera II Evis 180 GIF180 videogastroscope (Olympus, Tokyo, Japan) with high resolution (1,080 dpi), 1.5-fold magnification, and narrow-band imaging technology was used.

### 2.4. Histology

Histology was performed by a senior pathologist, who was aware of the endoscopic suspicion of esophageal squamous cell carcinoma. Biopsy specimens were immersed in formaldehyde for fixation and stained with hematoxylin and eosin. The lesions were classified in accordance with the Revised Vienna Classification [22]. In the absence of lamina propria invasion, noninvasive neoplastic lesions were divided into two groups based on the degree of intraepithelial neoplasia: low grade and high grade.

High-grade dysplasia, intraepithelial carcinoma, and carcinoma *in situ *were considered equivalent entities [21]. Whenever the lamina propria of the mucosa was invaded, the lesion was referred to as intramucosal carcinoma.

In this study, only findings of high-grade intraepithelial neoplasia (carcinoma *in situ) *and intramucosal carcinoma of squamous cells were considered true positives for esophageal squamous cell carcinoma [22].

### 2.5. Statistical Analysis

Values and 95% confidence intervals were calculated for sensitivity, specificity, positive predictive value, negative predictive value, accuracy, and positive and negative likelihood ratios.

## 3. Results

Of the 48 patients enrolled, five were excluded: one because he had an advanced malignant esophageal lesion that was easily detected by conventional endoscopy, three because they had esophageal stasis, and one because of a prior history of allergy to iodine. Of the remaining 43 patients, there were 14 men and 29 women. The median age was 59 years.

**Figure 1:**
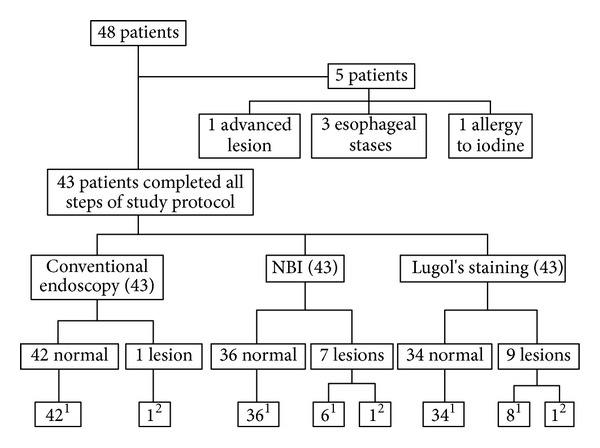
Flowchart of the study protocol. ^1^Total patients without squamous cell carcinoma (*n* = 42); ^2^total patients with squamous cell carcinoma (*n* = 1). NBI: Narrow band imaging.

**Figure 2:**
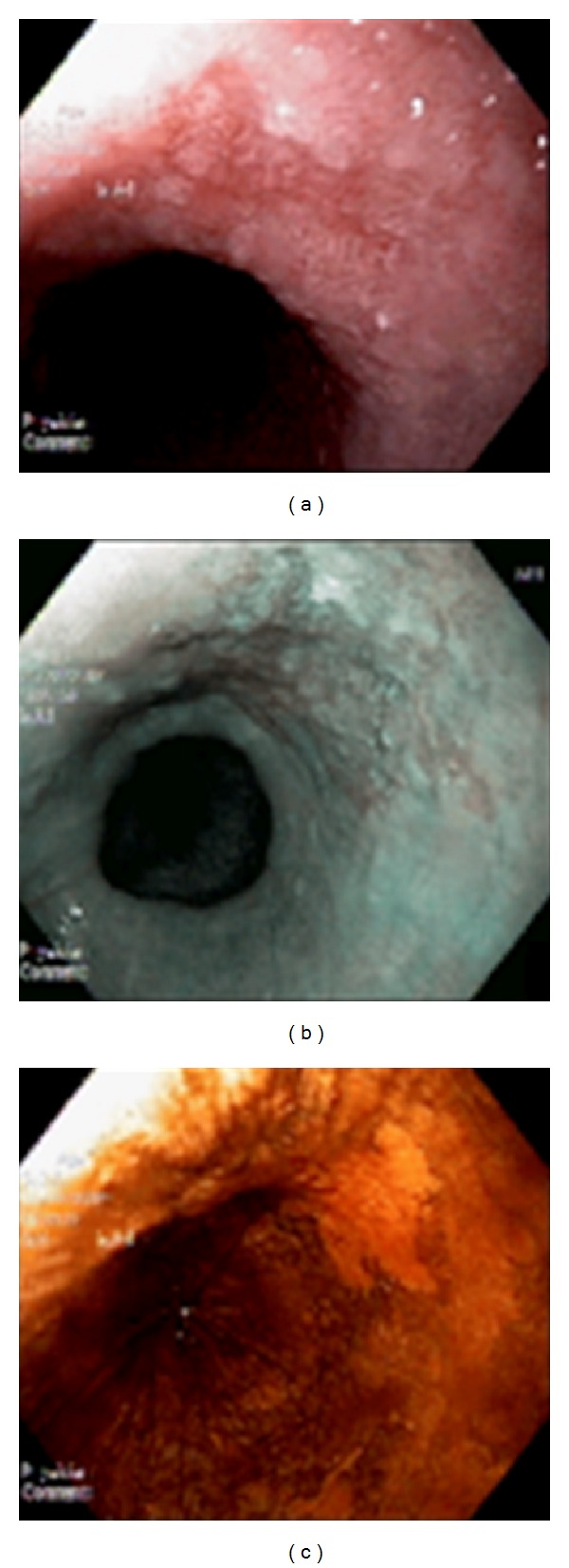
Esophageal lesion with flat morphology (0-IIb according to Paris Classification). (a) Conventional examination. (b) Narrow-band imaging. (c) Lugol's staining.

The 43 patients underwent all stages of the protocol of investigation for this study (Figure 1). Narrow-band imaging and Lugol's staining found seven and nine suspected lesions, respectively. Conventional endoscopy revealed one superficial lesion with a flat morphology (0-IIb according to Paris Classification), which was also detected by narrow-band imaging and Lugol's staining (Figure 2). This lesion proved to be an esophageal neoplasia (squamous cell carcinoma *in situ*), sized 15 mm in diameter, and was located in the thoracic esophagus. 

Of the seven lesions found by narrow-band imaging, histopathology revealed that one was normal mucosa, five were esophagitides, and one was squamous cell carcinoma *in situ*. Of the nine lesions found by Lugol's staining, histopathology revealed that eight were esophagitides and one was squamous cell carcinoma *in situ*. The same squamous cell carcinoma was found in one patient (Table 1).

The performance of narrow-band imaging was similar to that obtained by Lugol's staining. Sensitivity and negative predictive value were 100% for both methods, and the specificity was 85.7% (75.1%–96.3%) for narrow-band imaging and 81% (69.1%–92.8%) for Lugol's staining. Diagnostic performances for conventional endoscopic examinations, narrow-band imaging, and Lugol's staining are presented in Table 2.

In the Lugol's staining group, there were no cases of chemical laryngitis or hypersensitivity to iodine. No complications were reported with conventional or narrow-band imaging procedures.

## 4. Discussion

The present study selected patients with achalasia as an increased-risk group for esophageal squamous cell carcinoma. These patients usually present with delayed esophageal emptying, and they report worsening of these symptoms due to development of an obstructive tumor at late stages [23]. Without surveillance, esophageal carcinoma is usually diagnosed in advanced stages with poor prognosis [24].

The Lugol's staining technique is based on the presence of large amounts of glycogen in the squamous epithelium, which stains intensely with iodine; in contrast, dysplastic and carcinoma cells contain little or no glycogen, which results in no staining [15, 25–27]. Thus, upper gastrointestinal endoscopy with Lugol's staining is still considered the best method for the diagnosis and delimitation of superficial esophageal squamous cell carcinoma [27–29].

However, Lugol's solution irritates the mucosa and may lead to retrosternal chest pain and discomfort, because of its alcoholic nature. Its utilization is limited by other factors, namely, hypersensitivity to iodine and the risk of chemical esophagitis, laryngitis, and bronchopneumonia. Several authors have reported necrosis and injury to the esophageal and gastric mucosa caused by hypersensitivity to Lugol's solution [30, 31]. Furthermore, esophageal chromoendoscopy with Lugol's staining significantly increases the length of the examination period [32].

Narrow-band imaging enhances the visualization of superficial capillaries, as well as mucosal surface structure, and has an effect similar to that of chromoendoscopy. Moreover, narrow-band imaging does not have the limitations of Lugol's staining chromoendoscopy and could be considered as a potential alternative method for the detection of esophageal squamous cell carcinoma. 

Few studies have evaluated the capacity of narrow-band imaging without magnification to detect esophageal squamous cell carcinoma. Watanabe et al. [18] found that narrow-band imaging was more likely 2 folds than conventional white-light evaluation to detect pharyngeal squamous cell carcinoma. In a multicenter study that compared narrow-band imaging with conventional white-light evaluation, the accuracy was 90.2% and 55.3%, respectively (*P* < 0.0001) [33]. When Lugol's staining chromoendoscopy was compared with narrow-band imaging with image magnification, the sensitivity was the same (92.3%), but narrow-band imaging had a higher specificity (91.7% versus 72.2%) [34].

In our study, we compared Lugol's staining with narrow-band imaging technology without magnification. Many medical centers do not have the resources for magnification; therefore, the aim of this study was to determine whether narrow-band imaging alone would suffice to detect small and superficial neoplasias of the esophagus. Narrow-band imaging and Lugol's staining identified one esophageal neoplasia that was also detected by conventional white-light examination. Both methods had 100% sensitivity and negative predictive value. Although narrow-band imaging without magnification had a higher specificity for detecting early squamous cell neoplasias in the esophagus, it was similar to Lugol's staining: 85.7% (75.1%–96.3%) and 81% (69.1%–92.8%), respectively. In a study of patients with head and neck squamous cell carcinomas employing the same methodology, Ide et al. observed similar results when they compared narrow-band imaging without magnification with Lugol's staining [20]. Lee et al. and Takenaka et al. [33, 34] found that the sensitivity of narrow-band imaging for detecting esophageal squamous cell carcinoma and high-grade intraepithelial neoplasia was 90.9% (58.7%–99.8%), the specificity was 95.4% (90.3–98.3%), and the accuracy was 95.1% (90.1%–98.0%). 

Narrow-band imaging without magnification and Lugol's staining had equivalent performances; this indicates that narrow-band imaging is a potential surveillance method for patients with esophageal achalasia. 

Our study has limitations in its methodology. A sequential approach was adopted in which the standard endoscopy, narrow-band imaging, and the Lugol's staining were employed by the same operator in the same patient. This setting might have affected the results since the operator possessed prior information after each phase of endoscopic procedure. However, the sequential approach seems to be the best strategy for daily practice. Furthermore, this methodology was used in similar studies [31, 33, 34].

In conclusion, the results obtained with narrow-band imaging technology without magnification were comparable with those obtained with Lugol's staining for the screening of esophageal squamous cell carcinoma in patients with achalasia. Although narrow-band imaging does not have the risks and technical difficulties associated with Lugol's staining, larger multicenter studies are necessary in order to analyze the cost and benefits of this technology and to determine whether narrow-band imaging could replace Lugol's staining for screening of early-stage esophageal squamous cell carcinoma. 

## Figures and Tables

**Table 1:** Correlation between endoscopic findings and histopathologic examination by hematoxylin and eosin staining.

Method	Negative endoscopic findings	Positive endoscopic findings	Histopathologic examination		
			Squamous cell carcinoma (*in situ*)	Esophagitis	Normal
Conventional	42	1	1	—	—
Narrow-band imaging	36	7	1	5	1
Lugol's	34	9	1	8	0

**Table 2:** Comparison of the performance across methods (95% confidence interval) (%).

	Conventional examination	Narrow-band imaging	Lugol's
Sensitivity	100 (100-100)	100 (100-100)	100 (100-100)
Specificity	100 (100-100)	85.7 (75.1–96.3)	81 (69.1–92.8)
PPV	100 (100-100)	14.3 (−11.6–40.2)	11.1 (−9.4–31.6)
NPV	100 (100-100)	100 (100-100)	100 (100-100)
Accuracy	100 (100-100)	86 (75.7–96.4)	81.4 (69.8–93)
PLR	n/c	7	5.3
NLR	0	0	0

PPV: positive predictive value; NPV: negative predictive value; PLR: positive likelihood ratio; NLR: negative likelihood ratio; n/c: non calculable.
